# Relative transcript quantification by Quantitative PCR: Roughly right or precisely wrong?

**DOI:** 10.1186/1471-2199-6-10

**Published:** 2005-04-26

**Authors:** Rasmus Skern, Petter Frost, Frank Nilsen

**Affiliations:** 1 Marine Genome Biology, Institute of Marine Research, N-5817 Bergen, Norway

## Abstract

**Background:**

When estimating relative transcript abundances by quantitative real-time PCR (Q-PCR) we found that the results can vary dramatically depending on the method chosen for data analysis.

**Results:**

Analyses of Q-PCR results from a salmon louse starvation experiment show that, even with apparently good raw data, different analytical approaches [1,2] may lead to opposing biological conclusions.

**Conclusion:**

The results emphasise the importance of being cautious when analysing Q-PCR data and indicate that uncritical routine application of an analytical method will eventually result in incorrect conclusions. We do not know the extent of, or have a universal solution to this problem. However, we strongly recommend caution when analysing Q-PCR results e.g. by using two or more analytical approaches to validate conclusions. In our view a common effort should be made to standardise methods for analysis and validation of Q-PCR results.

## Communication

Reverse transcription (RT) followed by quantitative polymerase chain reaction (Q-PCR) is at present the most sensitive method for transcript abundance measurement. However, there are many sources of errors, both when purifying RNA, performing the RT reaction and during the PCR setup [3,4]. Q-PCR utilises optical measurement of generated amplicons to survey PCR amplifications. It is common to derive the initial template concentration from the number of amplification cycles required for a signal to reach a threshold chosen by the investigator [1,2,5]. In relative quantification the expression of a target gene is stated relative to a standard gene, which is assumed to be constitutively and uniformly expressed. One popular approach, the 2^-ΔΔCT ^method, assumes ≈100% efficient target and standard gene PCR reactions given that the results conform to certain criteria [1,5]. In recognition of the fact that PCR efficiencies may vary between runs or between target and standard genes, other numerous methods have emerged that calculate template concentrations using amplification simulations or PCR efficiencies derived from CT values or fluorescence data [2,6-9]. We here present the results of a case study showing that the interpretation of results may vary dramatically with the chosen method for data analysis.

**Figure 1:**
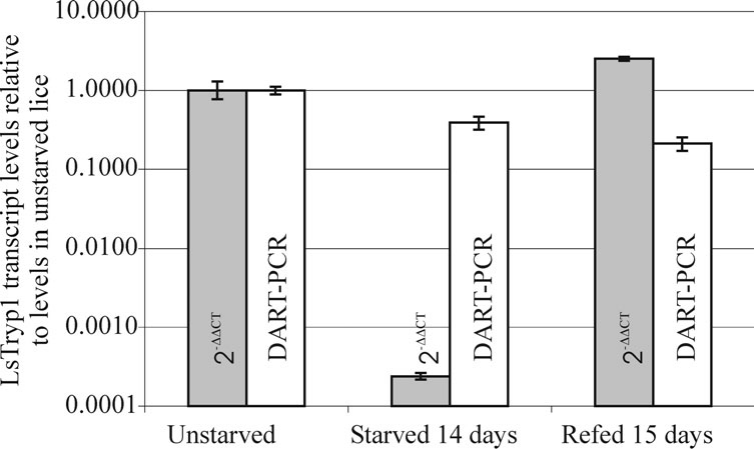
**Q-PCR analysis**. Transcript levels from the same Q-PCR runs analysed using the 2^-ΔΔCT ^method and the DART-PCR Excel Spreadsheet. Error bars indicate 95% confidence intervals.

We have analysed results from a salmon lice (*Lepeophtheirus salmonis*) starvation experiment using the 2^-ΔΔCT ^method [1] and the "DART method" adjusting for PCR efficiency differences [2]. When analysed using the 2^-ΔΔCT ^method, our results show that *LsTryp1 *transcript levels decrease following starvation and return to normal adult level when the louse subsequently gets access to food (Fig. 1). The inclinations obtained when plotting ΔCT or CT against log RNA concentration do not indicate significant differences in PCR efficiencies between *LsTryp1 *and *eEF1α *(Table 1). When analysed using the "DART method" the results indicate that *LsTryp1 *transcript levels decrease 2–3 fold when lice are starved and remain low when lice subsequently get access to food (Fig. 1). The PCR efficiencies, calculated from at least 3 points for each reaction [2], indicated significant differences in PCR efficiency between *eEF1α *and *LsTryp1 *in starved and refed lice but not in unstarved lice (Table 1).

By intuition it appears that surveying PCR efficiencies using several measured fluorescence points from each PCR reaction, as done using the "DART method", is superior to using one point from each reaction, as done when comparing ΔCT values using the 2^-ΔΔCT ^method. However, since PCR efficiencies calculated using the "DART method" exceed 100% in some instances, it is clear that this approach also has weaknesses. In the present example (Fig. 1) we would not have more confidence in one method than the other unless we had data from supplementary methods (e.g. microarrays) to support this. Consequently these data indicate that *LsTryp1 *transcript levels decrease when lice are starved, which is in accordance with the alleged digestive function of the encoded protein [10]. However, since the result varies between the "DART-method" and the 2^-ΔΔCT ^method, we are unable to determine how transcription is regulated after lice resume feeding. Thus, despite the fact that both the 2^-ΔΔCT ^method and the "DART-method" are theoretically sound given a number of assumptions [1,2], we may be mislead when these assumptions are not fulfilled.

All strategies for analysing Q-PCR data are based on a number of assumptions, and due to experimental errors none or few of these assumptions will be fulfilled entirely. Unfortunately, it is not always obvious when assumptions are broken to a degree that invalidates the conclusions. Since the sources of potential problems are diverse, no simple solution is available. Therefore we do not offer a universal analytical approach that can be applied to any given set of data and ensure a correct conclusion. Rather, we suggest investigators to urge caution when analysing results and hope that future discussions will lead to a more unified approach to Q-PCR data analysis and improved reliability of published results.

## Methods

Salmon lice (*Lepeophtheirus salmonis*) were reared as earlier described [10]. After development to the adult stage, 15 lice were removed with forceps from their anaesthetised (80 μg/ml benzocaine) salmon hosts (*Salmo salar*) and 3 lice were stored in RNA *later *(Ambion). The remaining 12 lice were starved in incubators with flowing seawater for 14 days. After starvation, 3 lice were sampled and stored as described above, and the remaining 9 lice were put in a tank with uninfected salmon where they could settle on their salmon hosts and resume feeding. After 15 days on their new hosts 3 lice were sampled and stored as described above. The experimental procedures were carried out in accordance with national regulations for use of animals in scientific research.

The transcript levels of *LsTryp1 *[10] and the reference gene *eEF1α *[11] in 1 selected unstarved, starved and refed lice were determined by quantitative real time PCR carried out with 3 parallels at 5 sequential 2-fold dilutions as previously described [10]. The RNA purification protocol is previously described [10] and cDNA syntheses were performed using MultiScribe™ according to the manufacturers recommendations (Applied Biosystems). The Q-PCR results were analysed by the 2^-ΔΔCT ^method as earlier described [10] and a method adjusting for PCR efficiency differences described by Peirson et al. [2]. The latter analysis was performed partially in the DART-PCR Excel spreadsheet [2]. When using the 2^-ΔΔCT ^method, at least 2 parallels were required at each dilution. Parallels were removed when the CT value differed more than 0.3 (CT<32) or 0.4 (CT = 32) from the most similar parallel at the same dilution. At least 4 dilutions were required for each stage. The resulting data were calibrated to unstarved lice and analysed as described by Kvamme et al.[10]. When using the "DART-method", dilutions were removed when PCR efficiency differed significantly (one way ANOVA, *α *= 0.05) from the other dilutions. The signal corresponding to the initial template concentration (R_0_) was derived using the average PCR efficiency for *LsTryp1 *and *eEF1α *when the PCR efficiencies were not significantly different (one way ANOVA, *α *= 0.05). When the PCR efficiency differed significantly, R_0 _was calculated using individual gene specific mean efficiencies. The mean R_0 _for each dilution of *LsTryp1 *was normalised to corresponding *eEF1α *values. The normalised R_0 _values were calibrated to the values for unstarved lice. 95% confidence intervals (CI) were derived from normalised R_0 _values.

## Authors' contributions

RS and PF conceived the study and designed the Q-PCR assay. RS carried out the assay and analyses, and wrote the first draft of the communication. PF contributed to development of the communication. FN provided expert input for the writing and supervised the study.

## Figures and Tables

**Table 1:** Quality assessment of the retrieved data. Quality assessment of the retrieved data. For the 2^-ΔΔCT ^results the table shows inclination and R^2 ^for ΔCT plotted against log RNA concentration and inclinations and R^2 ^for CT plotted against log RNA concentration for *eEF1α *and *LsTryp1*. For the DART-PCR results the table shows PCR-efficiencies for *eEF1α *and *LsTryp1 *calculated by DART-PCR and the p-value (one-way ANOVA) for the hypothesis that there is no difference between the efficiencies.

	2^-ΔΔCT ^results			DART-PCR results		
	ΔCT vs. log [RNA] (R^2^)	*eEF1α *CT vs. log [RNA] (R^2^)	*LsTryp1 *CT vs. log [RNA] (R^2^)	*eEF1α*	*LsTryp1*	ANOVA

Unstarved	-0.080 (0.16)	-2.7664 (0.95)	-2.7949 (0.99)	0.901	0.902	0.9863
Starved	0.065 (0.06)	-2.8815 (0.99)	-2.8171 (0.93)	0.839	0.491	0.0004
Refed	-0.052 (0.07)	-3.3109 (0.99)	-3.3845 (0.99)	0.883	1.095	0.0004
